# Factors Associated with Brachial-Ankle Pulse Wave Velocity in an Apparently Healthy Chinese Population

**DOI:** 10.1155/2020/9795240

**Published:** 2020-07-23

**Authors:** Liangmei Chen, Xiaomin Liu, Linpei Jia, Zheyi Dong, Qian Wang, Yizhi Chen, Yong Wang, Ying Zheng, Sasa Nie, KangKang Song, Delong Zhao, Shuwei Duan, Zuoxiang Li, Zhe Feng, Xuefeng Sun, Guangyan Cai, Weiguang Zhang, Xiangmei Chen

**Affiliations:** ^1^Department of Nephrology, Chinese PLA General Hospital, Chinese PLA Institute of Nephrology, State Key Laboratory of Kidney Diseases, National Clinical Research Center of Kidney Diseases, Beijing Key Laboratory of Kidney Disease, Beijing, China; ^2^Department of Nephrology, The Second Hospital of Jilin University, Changchun, China

## Abstract

**Purpose:**

To investigate the factors influencing brachial-ankle pulse wave velocity (baPWV) in an apparently healthy Chinese population, especially the associations between baPWV and indices of blood pressure (BP).

**Methods:**

A total of 1123 participants with no history of hypertension were enrolled in this study, and the baPWV and BP of all four limbs were measured along with other covariates. Correlation analyses and multivariate linear regression models were used to identify factors associated with baPWV.

**Results:**

A total of 1123 participants (male 43.3%, mean age: 58.4 ± 13.9 years) were included. The average baPWV was 14.87 ± 3.21 m/s, and no difference was found between the sexes. Age was positively correlated with baPWV (*r* = 0.65, *p* < 0.01), especially in females (*r* = 0.71 versus 0.56 in males). The correlation coefficient between age and baPWV increased markedly after the age of 65 years. In addition, the resting heart rate (RHR), waist-hip ratio, glomerular filtration rate, and plasma glucose level were significantly correlated with baPWV (*r* = 0.25, 0.22, -0.43, and 0.25, respectively; *p* < 0.01). BP parameters were highly positively correlated with baPWV, especially systolic BP (SBP) and pulse pressure (PP). Multivariate regression revealed that age, BP parameters, and RHR were independently correlated with baPWV (*p* < 0.01) after adjusting for confounding factors. The standardized coefficients of SBP were greater than those of PP, followed by diastolic BP (DBP).

**Conclusion:**

BaPWV increased with age, especially after 65 years. Age, BP, and RHR were independent factors associated with baPWV. The effect of SBP on baPWV was more prominent than that of PP.

## 1. Introduction

Arterial elasticity dysfunction is an important indicator of arteriosclerosis and is closely associated with cardiovascular diseases [1–3]. Increased arterial stiffness parallels structural changes in the medial layer of elastic arteries and is one of the pathological symptoms of vascular damage. Arterial stiffness is associated with future hypertension and cardiovascular events. Therefore, the prevention of arterial stiffness is of great importance [4].

Brachial-ankle pulse wave velocity (baPWV) is the most widely used measure of arterial stiffness in regular clinical and epidemiological settings due to its noninvasive and reproducible nature as well as the ease of follow-up and evaluation [5]. Higher baPWV was observed in patients with hypertension or other diseases, such as diabetes mellitus, dyslipidaemia, metabolic syndrome, chronic kidney disease, and ventricular hypertrophy [6–9]. baPWV was identified as an independent predictor of cardiovascular diseases, cardiovascular mortality, and all-cause mortality [1, 10].

Most studies on baPWV-associated factors have focused on patients with hypertension and the other diseases mentioned above [11–14], but arterial stiffness is not confined to patients with hypertension. In nonhypertensive individuals, it is not clear whether blood pressure (BP) has an effect on baPWV. The factors influencing baPWV have been widely studied, but this relationship is less well defined. Taking the resting heart rate (RHR) as an example, studies have shown a positive association with baPWV, no association, or even an inverse association [15]. However, numerous studies have demonstrated robust associations between baPWV and RHR, ageing, higher body mass index (BMI), and reduced glomerular filtration rate (GFR) [16–18]. These associations are not well studied in Chinese adults without hypertension. Furthermore, whether the increase in arterial stiffness with advancing age results from the age-associated increase in BP is unknown.

The present study measured baPWV and BP, including bilateral ankle/brachial systolic BP, bilateral ankle/brachial diastolic BP, and bilateral ankle/brachial pulse pressure (PP), in a healthy northern Chinese population without hypertension or other atherosclerotic factors. We investigated the factors associated with baPWV and particularly studied whether BP was an independent factor influencing baPWV.

## 2. Subjects and Methods

**Figure 1:**
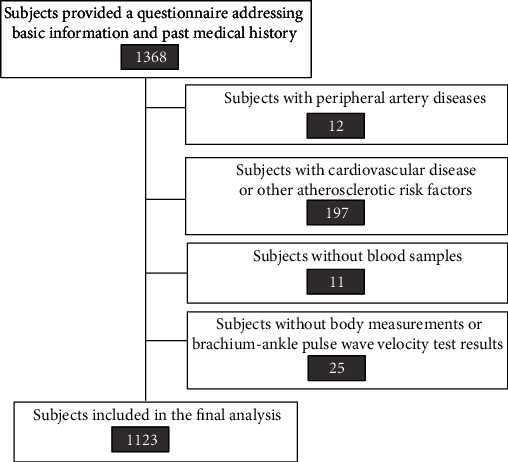
Flowchart of participant screening.

A total of 1368 participants were recruited among the residents of urban Beijing in 2014. All participants received an explanation of the purpose of this investigation and voluntarily provided their consent to participate in this study. All protocols were approved by the Ethics Committee of the Chinese PLA General Hospital. The exclusion criteria were as follows: (1) pregnant women; (2) individuals taking medication; (3) subjects with a medical history of peripheral vascular diseases; (4) subjects with hypertension (BP ≥ 140/90 mmHg) or diagnosed with chronic kidney disease, diabetes mellitus, or dyslipidaemia (total cholesterol ≥ 5.9 mmol/L, triglycerides ≥ 1.70 mmol/L); (5) subjects with a medical history of cardiovascular diseases, such as stroke and myocardial infarction; and (6) BMI ≥ 28.0 kg/m^2^ [17]. Data from 1123 participants were included in the final analysis (Figure 1).

Participants underwent a comprehensive assessment with an Artery Stiffness Detector (OMRON VP-1000), including measurements of bilateral baPWV and BP in all four limbs and electrocardiography. The examination was conducted after the participants rested in the supine position for at least 5 min, and all examinations were performed by the same senior physician at the Chinese PLA General Hospital. The average values of the bilateral baPWV and BP were used for the analysis. The sum-PP was the sum of the PPs in the four limbs. Physical measurements, including body height, weight, waist circumference, and hip circumference, were also taken. BMI was calculated as the weight (kg) divided by the height squared (m^2^). The waist-hip ratio (WHR) was calculated as the waist circumference divided by the hip circumference.

Subjects were required to fast for 8-12 h before blood sample collection. The plasma alanine aminotransferase (ALT), aspartate aminotransferase (AST), total protein (TP), albumin, total bilirubin (TB), plasma glucose (PG), urea, urea acid, creatinine, total cholesterol (TC), triglycerides (TG), high-density lipoprotein cholesterol (HDL-C), and low-density lipoprotein cholesterol (LDL-C) levels were measured at the Biochemistry Laboratory of the Chinese PLA General Hospital. The GFR was calculated according to the Chronic Kidney Disease Epidemiology Collaboration creatinine (CKD-EPI) equation.

Data are presented as the means ± standard deviations for continuous variables and as the percentages (%) for categorical variables. The population characteristics stratified by baPWV quartiles were compared using ANOVA (continuous normally distributed parameters), Kruskal-Wallis tests (continuous nonnormally distributed variables), or chi-square tests (categorical variables). Missing data was not imputed due to the small proportion (approximately 2%) of missing data.

Correlation analyses were used to determine the relationships between baPWV and variables (BP parameters, age, height, weight, BMI, waist circumference, etc.). The Pearson correlation analysis was used to determine relationships between normally distributed variables, and the Spearman correlation analysis was used for nonnormally distributed variables. Multivariate linear regression was used to identify factors affecting baPWV with “stepwise” as the variable filtering method. To avoid multicollinearity, we included only one BP parameter at a time as an independent variable in the regression model, with age, WHR, PG, GFR, and RHR as covariates. Data analyses were performed using SPSS version 25.0 for Mac (SPSS, Chicago, IL, USA).

## 3. Results

In total, 1123 participants were eligible for inclusion in this study, including 486 males (43.3%) and 637 females (56.7%). The age ranges of the males and females were both 21 to 86 years, and the mean age of the population was 58.4 ± 13.9 years. The average baPWV was 14.87 ± 3.21 m/s, and there was no difference between the sexes (male: 15.02 ± 2.84 m/s, female: 14.75 ± 3.46 m/s; *p* = 0.170). According to baPWV quartiles, we stratified the study population into four groups: Q1 (baPWV < 12.66 m/s), Q2 (12.66 ≤ baPWV < 14.36 m/s), Q3 (14.36 ≤ baPWV < 16.46 m/s), and Q4 (baPWV ≥ 16.46 m/s). The mean values of baPWV for the Q1-Q4 groups were 11.41 ± 0.98 m/s, 13.48 ± 0.50 m/s, 15.33 ± 0.58 m/s, and 19.26 ± 2.51 m/s, respectively.

There was a trend for gradually increasing age, GFR, RHR, and PG levels with increasing baPWV (*p* < 0.001) (Table 1). The highest age, GFR, and RHR were observed in individuals with the highest quartiles of baPWV. The mean value of the sum-PP was 246.6 ± 47.1 mmHg. All indices of BP increased significantly as baPWV increased.

**Figure 2:**
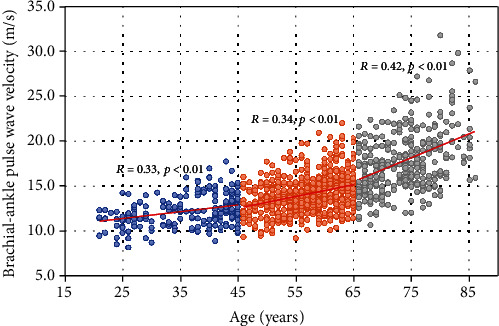
The scatter diagram between age and brachial-ankle pulse wave velocity in different age groups. Blue circles represent people aged ≤45 years, orange circles represent people aged between 46 and 65 years, and grey circles represent elderly people (>65 years).

The Pearson correlation coefficients between baPWV and other clinical variables are listed stratified by sex in Table 2. Age was positively correlated with baPWV (*r* = 0.65, *p* < 0.01), especially in females (*r* = 0.71 versus 0.56 in males). The correlation between age and baPWV became stronger after age 65, as shown in Figure 2. The GFR was negatively correlated with baPWV (*r* = −0.43, *p* < 0.01), especially in females (*r* = −0.48, *p* < 0.01). The WHR, RHR, and level of plasma glucose (PG) were significantly but weakly correlated with baPWV in the whole population (all 0.2 < *r* < 0.3, *p* < 0.01). In addition, height, WC, and the urea acid level were only weakly correlated with baPWV in females (all 0.2 < *r* ≤ 0.3, *p* < 0.01) (Table 2).

baPWV was positively correlated with all parameters of BP, especially SBP and sum-PP. The correlation coefficient of SBP (*r* ≥ 0.57) was greater than that of sum-PP (*r* = 0.53) in males, followed by PP (*r* ≤ 0.50) and DBP (*r* ≤ 0.44). Except for left brachial SBP (*r* = 0.70), the correlation coefficients of SBP (*r* ≤ 0.68) were smaller than those of sum-PP (*r* = 0.69) in females, followed by PP (*r* ≤ 0.66) and DBP (*r* ≤ 0.44) (Table 2).

To analyse the relationships between baPWV and the BP indices, stepwise multivariate linear regression analysis was performed; the results are shown in Table 3. After adjusting for confounding factors (including WHR, PG, GFR, age, and RHR), the parameters of BP (sum-PP, SBP, DBP, and PP) were stable and significant determinants of baPWV. The standardized coefficient of SBP was greater than that of sum-PP in males, but the opposite was true in females, both followed by PP and DBP. In addition to the BP parameters, age and the RHR were also independent factors influencing baPWV.

## 4. Discussion

The present cross-sectional study of an apparently healthy northern Chinese population showed that baPWV increases with age and that the rate of increase accelerates after 65 years of age. People with higher RHRs or BP levels tend to have higher baPWV. The significantly positive correlation of BP with baPWV indicates that a higher BP is still a contributing factor to arterial stiffness, even when it is within the normal range. The effect of SBP on baPWV was more prominent than that of PP.

BaPWV is widely used to evaluate arterial stiffness, which reflects alterations in the structural and functional properties of the central and peripheral arteries. Carotid-femoral PWV (cfPWV) is the most validated technique and is regarded as the gold standard for PWV measurement [19]. However, because of its methodological difficulties and limited availability, cfPWV is not generally implemented in China. In contrast, the measurement of baPWV is easy and reproducible, as it simply involves using BP cuffs on the four extremities.

baPWV is elevated in patients with diabetes mellitus, hypertension, metabolic syndrome, chronic kidney disease, and sleep apnoea syndrome; and ageing, tachycardia, and the postmenopause period also result in increases in baPWV [19–22]. baPWV is an independent risk factor for cardiovascular events in patients with hypertension, but the optical cut-off values of baPWV for predicting cardiovascular disease are still controversial. For example, Ohkuma et al. proposed 18.3 m/s [23], while Kawai et al. suggested 17.5 m/s [24, 25]. In the general Chinese population, Lu et al. reported that a baPWV of 16.7 m/s is the optimal threshold for cardiovascular risk stratification [26].

With the increasing use of baPWV to measure arterial stiffness and predict the risks of arteriosclerosis and cardiovascular death, the amount of research on the factors associated with baPWV in various populations has increased. Many studies have reported that baPWV increases substantially with increasing age [27–29], and our results are consistent with these well-established findings. A study including 3215 Japanese adolescents claimed that the effect of ageing was more prominent in males than in females [29]. However, the sex difference seems to be the opposite in adults, which has also been validated in our study (*r* = 0.56 in male versus *r* = 0.71 in female, *p* < 0.001) and other studies [17, 28]. The level of plasma oestrogen or androgen during menopause may explain the augmented increase in baPWV with ageing in females [17, 30]. Furthermore, our study illustrated a linear relationship between age and the value of baPWV, and a relatively closer correlation was observed in elderly people. A study reported that the age-related progression of arterial stiffness could be explained by a growth curve rather than a straight line [16].

Our study demonstrated that a higher BP is a contributing factor to arterial stiffness, even when it is within the normal range, and the effect of SBP on baPWV was more prominent than that of PP. The association between BP and arterial stiffness has been debated in various studies [11, 13, 26, 27, 31, 32]. Longitudinal studies have shown that a large PP in childhood plays a role in the development of subclinical vascular damage in adulthood, and the early prevention of a large PP can reduce the future risk of cardiovascular disease [14]. Some BP-derived indicators, such as long-term BP variability and sum-PP [5, 33], were also reported to be significantly associated with baPWV. This relationship between BP and arterial stiffness may be bidirectional because arterial stiffness contributes to the increase in BP, and arterial stiffening is accelerated in patients with hypertension [34]. They behave reciprocally as cause and effect, interacting in a vicious cycle [35]. However, a statement from the American Heart Association said that arterial stiffness represents a cause rather than a consequence of hypertension [15]. Monitoring BP may be useful for the identification of vascular impairment [36], and baPWV can be used to predict the progression of BP and incident hypertension and determine the individual response to antihypertensive treatment [37–40]. Whether baPWV could be a useful screening tool to identify apparently healthy subjects who should be targeted for inventions aimed at preventing incident hypertension requires further study.

The effects of the RHR on PWV are still controversial, with conflicting results being observed. Some researchers in the Corinthia study suggested that the influence of the RHR on PWV is mediated by BP [41], and the product of the RHR times the BP was reported to be associated with baPWV [42]. However, approximately half of the existing epidemiological studies have reported a significant BP-independent association between the RHR and PWV [43]. Our study demonstrated that an increased RHR is independently associated with a higher baPWV, regardless of other confounders in apparently healthy individuals. Regarding the mechanism, an increased RHR can change the viscoelasticity of the arterial wall, thus increasing arterial stiffness [43]. A study including 912 females showed that the urea acid level and baPWV had a positive nonlinear correlation [44], but our study showed that the level of urea acid was significantly linearly correlated with baPWV in females but not in males. In the previous literature, total homocysteine levels, BMI, low creatinine clearance, proteinuria, urine albumin, and salt intake were also reported to be associated with baPWV [22, 38, 45].

The results of this study should be interpreted in light of potential limitations. Although the study participants included people without hypertension or other cardiovascular diseases, the BP measurements were only obtained on a single occasion and the medical history was not reliable enough. Our study is cross-sectional; therefore, we could not determine the temporality of the associated factors, and we could not identify a causal relationship between baPWV and BP. These problems should be addressed in future longitudinal studies with larger populations.

## 5. Conclusions

In summary, the subjects included in our study were apparently healthy and without cardiovascular risk factors, and we verified the factors affecting baPWV and the association between BP and baPWV. The results showed that in addition to age and the RHR, BP was independently associated with baPWV. The development of arterial stiffness accelerates in elderly people. The standardized coefficients of SBP were greater than those of PP, followed by DBP. These findings underscore the importance of early monitoring of higher SBP to protect vascular elasticity.

## Figures and Tables

**Table 1:** Demographic and clinical characteristics of the participants.

Characteristics	Total (*N* = 1123)	baPWV quartiles (m/s)				*p* value
		Q1 (<12.66) (*N* = 281)	Q2 (12.66-14.36) (*N* = 281)	Q3 (14.36-16.46) (*N* = 281)	Q4 (≥16.46) (*N* = 280)	
Male, *N* (%)	486 (43.3)	98 (34.9)	137 (48.8)	132 (47.0)	119 (42.5)	0.004
Age (years)	58.4 ± 13.9	46.4 ± 13.2	55.1 ± 10.8^∗^	61.6 ± 10.0^∗^^&^	70.6 ± 8.4^∗^^& #^	<0.001
Physical measurements						
Height (cm)	164.8 ± 8.0	165.4 ± 7.5	165.8 ± 8.5	165.5 ± 7.5	162.6 ± 8.0^∗^^& #^	<0.001
Weight (kg)	66.2 ± 11.5	64.5 ± 12.0	67.8 ± 11.9^∗^	67.7 ± 10.4^∗^	64.8 ± 11.1^& #^	<0.001
BMI (kg/m^2^)	24.3 ± 3.3	23.5 ± 3.4	24.5 ± 3.1^∗^	24.7 ± 3.1^∗^	24.4 ± 3.3^∗^	<0.001
WC (cm)	85.6 ± 10.4	81.3 ± 11.0	86.1 ± 10.0^∗^	87.5 ± 9.6^∗^	87.5 ± 9.9^∗^	<0.001
HC (cm)	98.2 ± 6.8	96.8 ± 6.9	98.4 ± 6.6	99.0 ± 6.5^∗^	98.6 ± 7.3	0.001
WHR	0.87 ± 0.07	0.84 ± 0.07	0.87 ± 0.06^∗^	0.88 ± 0.08^∗^	0.89 ± 0.06^∗^	<0.001
RHR	67.2 ± 10.1	65.1 ± 8.9	65.6 ± 8.9	67.7 ± 10.3^∗^^&^	70.4 ± 11.2^∗^^& #^	<0.001
baPWV (m/s)	14.87 ± 3.21	11.41 ± 0.98	13.48 ± 0.50	15.33 ± 0.58	19.26 ± 2.51	<0.001
Biochemical examination						
ALT (U/L)	20.6 ± 13.4	20.0 ± 16.3	21.8 ± 13.1	20.9 ± 13.2	19.7 ± 10.1	0.242
AST (U/L)	20.8 ± 8.4	19.3 ± 7.6	21.2 ± 8.3^∗^	21.4 ± 9.1^∗^	21.2 ± 8.2^∗^	0.007
AST/ALT ratio	1.13 ± 0.36	1.13 ± 0.36	1.09 ± 0.37	1.13 ± 0.37	1.18 ± 0.35^&^	0.027
TP (g/L)	70.9 ± 3.8	70.7 ± 3.8	70.8 ± 4.0	71.3 ± 3.8	71.0 ± 3.7	0.190
Albumin (g/L)	45.1 ± 2.9	45.4 ± 2.4	45.1 ± 2.5	44.9 ± 3.5^∗^	44.9 ± 3.1^∗^	0.109
TB (*μ*mol/L)	12.1 ± 5.1	12.0 ± 4.9	11.7 ± 4.2	12.1 ± 5.5	12.4 ± 5.6	0.419
PG (mmol/L)	5.4 ± 1.2	5.0 ± 0.9	5.3 ± 0.9^∗^	5.5 ± 1.2^∗^^&^	5.9 ± 1.4^∗^^& #^	<0.001
Urea (mmol/L)	5.1 ± 1.4	4.6 ± 1.3	5.1 ± 1.4^∗^	5.2 ± 1.4^∗^	5.4 ± 1.2^∗^^&^	<0.001
Creatinine (*μ*mol/L)	71.1 ± 16.5	66.8 ± 12.4	71.5 ± 21.7^∗^	72.8 ± 14.7^∗^	73.0 ± 15.1^∗^	<0.001
Uric acid (*μ*mol/L)	318.5 ± 79.1	296.5 ± 83.6	318.4 ± 74.8	334.1 ± 75.5^∗^	325.1 ± 77.6^∗^	<0.001
TC (mg/mL)	4.6 ± 0.9	4.4 ± 0.8	4.6 ± 0.9^∗^	4.7 ± 1.0^∗^	4.6 ± 0.9^#^	0.001
TG (mg/mL)	1.4 ± 0.9	1.2 ± 0.7	1.4 ± 0.9^∗^	1.5 ± 1.0^∗^^&^	1.4 ± 0.9^∗^^#^	<0.001
HDL-C (mg/mL)	1.4 ± 0.4	1.4 ± 0.4	1.3 ± 0.3	1.3 ± 0.4^∗^	1.4 ± 0.4	0.101
TC/HDL ratio	3.6 ± 1.1	3.4 ± 1.2	3.6 ± 1.1^∗^	3.7 ± 1.2^∗^	3.5 ± 1.0^#^	0.003
LDL-C (mg/mL)	2.8 ± 0.8	2.7 ± 0.8	2.9 ± 0.8	2.9 ± 0.8	2.8 ± 0.8	<0.001
GFR (mL/min/1.73m^2^)	92.7 ± 18.6	103.7 ± 16.9	96.1 ± 18.4^∗^	88.9 ± 16.0^∗^^&^	82.2 ± 15.7^∗^^& #^	<0.001
Parameters of BP (mmHg)						
Sum-PP	246.6 ± 47.1	210.4 ± 29.6	231.7 ± 34.3^∗^	255.0 ± 38.2^∗^^&^	289.6 ± 43.8^∗^^& #^	<0.001
Brachial SBP	126.9 ± 14.9	112.8 ± 12.3	123.2 ± 10.0^∗^	131.7 ± 11.0^∗^^&^	140.1 ± 10.4^∗^^& #^	<0.001
Brachial DBP	74.9 ± 9.3	67.8 ± 7.9	74.8 ± 8.5^∗^	78.2 ± 8.6^∗^^&^	78.9 ± 7.8^∗^^&^	<0.001
Brachial PP	52.1 ± 10.2	45.3 ± 6.7	48.4 ± 7.6^∗^	53.5 ± 8.7^∗^^&^	61.1 ± 9.7^∗^^& #^	<0.001
Ankle SBP	143.2 ± 20.8	124.0 ± 15.6	138.7 ± 15.6^∗^	149.8 ± 15.7^∗^^&^	160.1 ± 16.8^∗^^& #^	<0.001
Ankle DBP	72.2 ± 9.4	64.8 ± 7.8	71.8 ± 7.9^∗^	75.9 ± 8.2^∗^^&^	76.4 ± 8.6^∗^^&^	<0.001
Ankle PP	71.0 ± 15.9	59.4 ± 10.6	66.9 ± 12.6^∗^	73.9 ± 13.1^∗^^&^	83.7 ± 15.8^∗^^& #^	<0.001

^∗^ *p* < 0.05 compared with Q1. ^&^*p* < 0.05 compared with Q2. ^#^*p* < 0.05 compared with Q3. baPWV: brachial-ankle pulse wave velocity; BMI: body mass index; WC: waist circumference; HC: hip circumference; WHR: waist-hip ratio; ALT: alanine aminotransferase; AST: aspartate aminotransferase; TP: total protein; TB: total bilirubin; PG: plasma glucose; TC: total cholesterol; TG: triglycerides; HDL-C: high-density lipoprotein cholesterol; LDL-C: low-density lipoprotein cholesterol; GFR: glomerular filtration rate; RHR: resting heart rate; BP: blood pressure; SBP: systolic BP; DBP: diastolic BP; PP: pulse pressure.

**Table 2:** Correlation analysis of brachial-ankle pulse wave velocity stratified by sex.

Parameters	Total (*n* = 1123)	Male (*n* = 486)	Female (*n* = 637)
Age (years)	0.65^∗^	0.56^∗^	0.71^∗^
Physical measurements			
Height (cm)	-0.14	-0.20	-0.28^#^
Weight, kg	-0.03	-0.12	-0.01
BMI (kg/m^2^)	0.07	-0.04	0.12
WC (cm)	0.18	0.06	0.24^#^
HC (cm)	0.07	-0.04	0.12
WHR	0.22^#^	0.15	0.27^#^
RHR	0.25^#^	0.28^#^	0.24^#^
Biochemical examination			
ALT (U/L)	-0.03	-0.11	0.03
AST (U/L)	0.07	-0.03	0.12
AST/ALT ratio	0.08	0.16	0.05
TP (g/L)	0.03	0.05	0.03
Albumin (g/L)	-0.09	-0.11	-0.09
TB (*μ*mol/L)	0.02	0.07	-0.03
PG (mmol/L)	0.25^#^	0.22^#^	0.28^#^
Urea (mmol/L)	0.19	0.01	0.30^∗^
Creatinine (*μ*mol/L)	0.14	0.18	0.12
Uric acid (*μ*mol/L)	0.13	0.01	0.21^#^
TC (mg/mL)	0.05	0.06	0.05
TG (mg/mL)	0.10	0.08	0.11
HDL-C (mg/mL)	-0.01	0.03	-0.02
TC/HDL ratio	0.03	0.02	0.03
LDL-C (mg/mL)	0.04	0.04	0.04
GFR (mL/min/1.73m^2^)	-0.43^∗^	-0.39^∗^	-0.48^∗^
Sum-PP	0.63^∗^	0.53^∗^	0.69^∗^
Brachial SBP	0.66^∗^	0.62^∗^	0.68^∗^
Brachial DBP	0.41^∗^	0.40^∗^	0.43^∗^
Brachial PP	0.58^∗^	0.50^∗^	0.66^∗^
Ankle SBP	0.62^∗^	0.57^∗^	0.66^∗^
Ankle DBP	0.42^∗^	0.44^∗^	0.41^∗^
Ankle PP	0.57^∗^	0.47^∗^	0.63^∗^

^∗^ *r* > 0.3, *p* < 0.01; ^#^*r* > 0.2, *p* < 0.01. baPWV: brachial-ankle pulse wave velocity; BMI: body mass index; WC: waist circumference; HC: hip circumference; WHR: waist-hip ratio; ALT: alanine aminotransferase; AST: aspartate aminotransferase; TP: total protein; TB: total bilirubin; PG: plasma glucose; TC: total cholesterol; TG: triglycerides; HDL-C: high-density lipoprotein cholesterol; LDL-C: low-density lipoprotein cholesterol; GFR: glomerular filtration rate; RHR: resting heart rate; BP: blood pressure; SBP: systolic BP; DBP: diastolic BP; PP: pulse pressure.

**Table 3:** Coefficients of variables in multivariate linear regression analyses.

BP indices as independent variables		Unstandardized coefficients (95% confidence interval)			Standardized coefficients		
		Age	BP indices	RHR	Age	BP indices	RHR
Total	Sum-PP	9.8 (8.8, 10.8)	2.8 (2.5, 3.1)	8.2 (7.0, 9.4)	0.42	0.41	0.26
	Brachial SBP	10.2 (9.2, 11.1)	9.7 (8.7, 10.6)	5.6 (4.4, 6.8)	0.45	0.42	0.18
	Brachial DBP	13.9 (13.0, 14.9)	8.7 (7.3, 10.2)	5.6 (4.2, 6.9)	0.60	0.25	0.17
	Brachial PP	11.2 (9.8, 12.6)	11.5 (10.1, 12.9)	8.3 (7.0, 9.5)	0.48	0.36	0.26
	Ankle SBP	10.7 (9.8, 11.7)	6.3 (5.7, 6.9)	7.5 (6.4, 8.7)	0.46	0.41	0.24
	Ankle DBP	13.8 (12.9, 14.7)	9.5 (8.1, 10.9)	6.4 (5.2, 7.7)	0.60	0.28	0.20
	Ankle PP	10.9 (9.8, 11.9)	7.1 (6.2, 7.9)	8.2 (6.9, 9.4)	0.47	0.35	0.26

Male	Sum-PP	8.6 (7.2, 10.0)	2.5 (2.1, 3.0)	9.1 (7.4, 10.8)	0.42	0.40	0.34
	Brachial SBP	8.9 (7.6, 10.1)	9.8 (8.5, 11.2)	5.6 (4.1, 7.3)	0.43	0.45	0.21
	Brachial DBP	11.3 (10.0, 12.7)	9.7 (7.4, 11.9)	5.1 (3.2, 7.0)	0.55	0.30	0.19
	Brachial PP	8.9 (7.5, 10.3)	11.2 (9.1, 13.3)	9.1 (7.4, 10.8)	0.44	0.36	0.33
	Ankle SBP	8.8 (7.5, 10.1)	6.0 (5.1, 6.9)	7.9 (6.3, 9.5)	0.43	0.42	0.29
	Ankle DBP	11.2 (9.9, 12.4)	11.0 (9.0, 13.0)	6.1 (4.4, 7.9)	0.54	0.36	0.23
	Ankle PP	9.2 (7.8, 10.6)	6.0 (4.8, 7.3)	9.0 (7.3, 10.8)	0.45	0.33	0.33

Female	Sum-PP	10.9 (9.5, 12.4)	3.0 (2.6, 3.4)	8.3 (6.6, 9.9)	0.44	0.42	0.23
	Brachial SBP	12.1 (10.6, 13.5)	9.2 (7.9, 10.6)	5.7 (4.0, 7.4)	0.51	0.39	0.16
	Brachial DBP	16.1 (14.8,17.4)	7.4 (5.2, 9.5)	5.8 (3.9, 7.7)	0.65	0.19	0.16
	Brachial PP	13.6 (11.7, 15.5)	12.0 (10.0, 13.9)	8.0 (6.3, 9.7)	0.55	0.37	0.22
	Ankle SBP	12.9 (11.5, 14.3)	6.3 (5.4, 7.3)	7.2 (5.6, 8.9)	0.52	0.39	0.20
	Ankle DBP	16.6 (15.2, 18.0)	8.2 (6.1, 10.2)	6.5 (4.6, 8.3)	0.67	0.21	0.18
	Ankle PP	12.6 (11.2, 14.0)	7.6 (6.4, 8.8)	8.2 (6.5, 9.9)	0.51	0.35	0.23

RHR: resting heart rate; BP: blood pressure; SBP: systolic BP; DBP: diastolic BP; PP: pulse pressure.

## Data Availability

The data used to support the findings of this study are available from the corresponding author upon reasonable request.
